# Supplementary material for: Delivery by caesarean section and risk of childhood obesity: analysis of a Peruvian prospective cohort
Source: PeerJ. 2015 Jun 23;3:e1046. doi: 10.7717/peerj.1046 (PMC4485704; doi:10.7717/peerj.1046)
Supplement: Supplemental Information 1 [file peerj-03-1046-s001.docx]

## Supplementary Figure 1. Flowchart of the participants included in the prospective analysis. The Young Lives Study, Younger Cohort, Peru.

Excluded because of prematurity (n=549)

Excluded because of missing values (n=529)

Potential Participants (n=1,503)

Younger Cohort (n=2,052)

Included at Baseline (n=974)

Overweight (n=132)

Obesity (n=34)

Overnutrition (n=166)

Outcome: Obesity

Incidence Analysis (n=940)

Outcome: Overweight

Incidence Analysis (n=808)

Outcome: Overnutrition

Incidence Analysis (n=808)

## Supplementary Figure 2: Time at which variables were assessed and number of children-years included in the analysis according to each outcome.


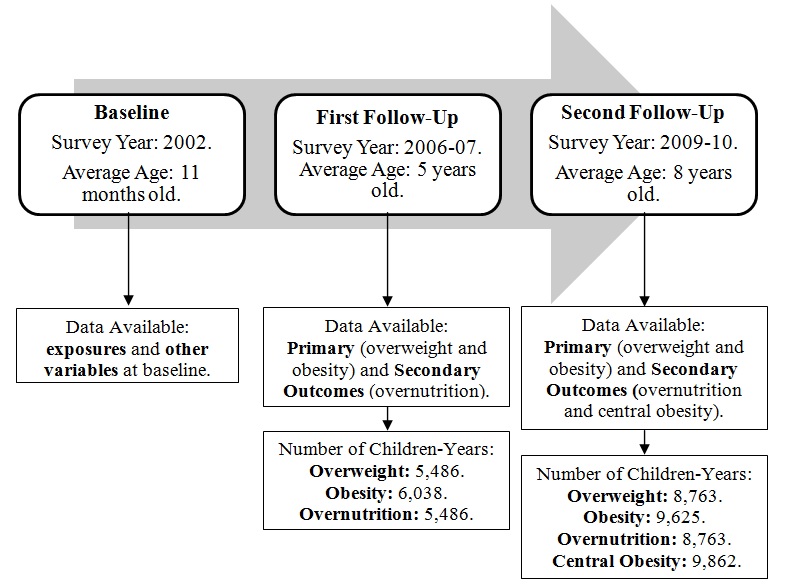


## Supplementary Table 1. Excluded and included participants due to missing values. The Young Lives Study, Younger Cohort, Baseline, Peru.

| Variable | Excluded (%) | Included (%) | p¥ |
| --- | --- | --- | --- |
| ***Mothers*** |  |  |  |
| Maternal BMI | n=516 | n=956 |  |
| Normal Weight | 72.09 | 54.71 |  |
| Overweight | 23.45 | 34.73 | <0.001 |
| Obesity | 4.46 | 10.56 |  |
| Maternal Education | n=497 | n=926 |  |
| None/Primary | 79.28 | 33.91 |  |
| High School | 17.51 | 43.84 | <0.001 |
| Higher Education | 3.22 | 22.25 |  |
| Wealth Index | n=528 | n=973 |  |
| Bottom | 61.36 | 22.92 |  |
| Middle | 31.82 | 35.15 | <0.001 |
| Top | 6.82 | 41.93 |  |
| Location | n=529 | n=974 |  |
| Urban | 38.00 | 79.57 |  |
| Rural | 62.00 | 20.43 | <0.001 |
| ***Children*** |  |  |  |
| Gender | n=529 | n=974 |  |
| Male | 46.31 | 50.10 |  |
| Female | 53.69 | 49.90 | 0.160 |
| Age | n=529 | n=974 |  |
| <1 year | 49.15 | 48.15 |  |
| ≥1 year | 50.85 | 51.85 | 0.712 |
| Birth Weight | n=343 | n=927 |  |
| 2500-4000 | 89.21 | 91.26 |  |
| ≤2500 | 5.54 | 2.27 | 0.010 |
| ≥4000 | 5.25 | 6.47 |  |
| Breastfeeding | n=527 | n=961 |  |
| Yes | 99.81 | 99.27 |  |
| No | 0.19 | 0.73 | 0.174 |
| BMI | n=503 | n=964 |  |
| Normal Weight | 91.65 | 87.14 |  |
| Overweight | 6.16 | 10.48 | 0.022 |
| Obesity | 2.19 | 2.39 |  |
| Hospitalised after birth | n=527 | n=961 |  |
| Yes | 1.52 | 9.47 |  |
| No | 98.48 | 90.53 | <0.001 |
| Birth Order | n=516 | n=947 |  |
| 1st | 18.99 | 42.13 |  |
| 2nd | 19.38 | 26.50 | <0.001 |
| ≥3rd | 61.63 | 31.36 |  |
| Delivery | n=4 | n=974 |  |
| No Caesarean section | 100.00 | 84.39 |  |
| Caesarean section | 0.00 | 15.61 | 0.390 |

¥Chi2 test

## Supplementary Table 2. Sociodemographic characteristics of both mothers and children according to children nutritional status. The young lives study, Younger Cohort, Baseline, Peru.

|  | Normal Weight (%) | Overweight (%) | Obesity (%) | p¥ |
| --- | --- | --- | --- | --- |
| ***Mothers*** |  |  |  |  |
| Maternal BMI | n=823 | n=100 | n=23 |  |
| Normal Weight | 56.14 | 46.00 | 34.78 |  |
| Overweight | 33.90 | 41.00 | 43.48 | 0.069 |
| Obesity | 9.96 | 13.00 | 21.74 |  |
| Maternal Education | n=798 | n=95 | n=23 |  |
| None/Primary | 35.84 | 16.84 | 34.78 |  |
| High School | 43.23 | 51.58 | 43.48 | 0.005 |
| Higher Education | 20.93 | 31.58 | 21.74 |  |
| Wealth Index | n=839 | n=101 | n=23 |  |
| Bottom | 24.31 | 9.90 | 17.39 |  |
| Middle | 35.76 | 30.69 | 30.43 | 0.001 |
| Top | 39.93 | 59.41 | 52.17 |  |
| Location | n=840 | n=101 | n=23 |  |
| Urban | 77.74 | 94.06 | 82.61 |  |
| Rural | 22.26 | 5.94 | 17.39 | 0.001 |
| ***Children*** |  |  |  |  |
| Gender | n=840 | n=101 | n=23 |  |
| Male | 49.88 | 52.48 | 43.48 |  |
| Female | 50.12 | 47.52 | 56.52 | 0.725 |
| Age | n=840 | n=32 | n=23 |  |
| <1 year | 45.24 | 68.32 | 69.57 |  |
| ≥1 year | 54.76 | 31.68 | 30.43 | <0.001 |
| Birth Weight | n=798 | n=97 | n=22 |  |
| 2500-4000 | 91.10 | 93.81 | 86.36 |  |
| ≤2500 | 2.63 | 0.00 | 0.00 | 0.279 |
| ≥4000 | 6.27 | 6.19 | 13.64 |  |
| Breastfeeding | n=830 | n=98 | n=23 |  |
| Yes | 99.16 | 100.00 | 100.00 |  |
| No | 0.84 | 0.00 | 0.00 | 0.598 |
| Hospitalized after birth | n=830 | n=98 | n=23 |  |
| Yes | 9.52 | 9.18 | 8.70 |  |
| No | 90.48 | 90.82 | 91.30 | 0.986 |
| Birth Order | n=816 | n=100 | n=22 |  |
| 1st | 41.05 | 50.00 | 45.45 |  |
| 2nd | 26.72 | 24.00 | 31.82 | 0.423 |
| ≥3rd | 32.23 | 26.00 | 22.73 |  |
| Delivery | n=840 | n=101 | n=23 |  |
| No Caesarean section | 85.60 | 76.24 | 69.57 |  |
| Caesarean section | 14.40 | 23.76 | 30.43 | 0.008 |

¥Chi2 test
